# Last-minute cancellation of elective lung cancer surgery is associated with poorer survival

**DOI:** 10.1093/icvts/ivae172

**Published:** 2024-10-08

**Authors:** Marco Nardini, Nilanjan Chaudhuri, Joshil Lodhia, Richard Milton, Peter Tcherveniakov, Elaine Teh, Alessandro Brunelli

**Affiliations:** Department of Thoracic Surgery, St James’s University Hospital, Leeds, UK; Department of Thoracic Surgery, St James’s University Hospital, Leeds, UK; Department of Thoracic Surgery, St James’s University Hospital, Leeds, UK; Department of Thoracic Surgery, St James’s University Hospital, Leeds, UK; Department of Thoracic Surgery, St James’s University Hospital, Leeds, UK; Department of Thoracic Surgery, St James’s University Hospital, Leeds, UK; Department of Thoracic Surgery, St James’s University Hospital, Leeds, UK; School of Medicine, University of Leeds, Leeds, UK

**Keywords:** Lung cancer, Surgery, Lung resection, Cancellation, Outcome, Survival

## Abstract

**OBJECTIVES:**

Our objective was to assess the incidence and reason of last-minute cancellations before surgery for lung cancer and their association with outcomes.

**METHODS:**

Retrospective analysis on all consecutive patients booked for elective lung cancer surgery from January 2017 through December 2022 in a single centre. Last-minute cancellation: a cancellation occurring within the last 24 h from the planned operation. Cancellation categories: process-related and patient-related. The short-term and long-term outcomes of patients who experienced a last-minute cancellation were analysed.

**RESULTS:**

197 patients of 1587 (12%) had a last-minute cancellation: 156 (79%) were process-related and 41 (21%) were patient-related. Three percent (5/156) of patients cancelled for process reasons did not receive surgery versus 39% (16/41) of those cancelled for patient-related reasons, *P* < 0.0001. The 90-day mortality rates of cancelled and non-cancelled patients were similar (4.6% vs 4.7%, *P* = 1). Five-year overall survival of patients with cancellation was 58% (95% confidence interval 49–66) vs 69% (95% confidence interval 66–71) of those without cancellations, *P* = 0.022. Among those who had a cancellation, the 5-year overall survival of those with process-related cancellations was 61% (52–60) vs 35% (14–58) of those with patient-related cancellations (adjusted *P* value for multiple comparisons = 0.14). Cox regression analysis showed that surgery cancellations within the last 24 h for patient-related (hazard ratio 1.87, 95% confidence interval 1.02–3.42, *P* = 0.043) reasons remained a factor associated with poorer overall survival after adjusting for clinical stage, type of operation and patient-related variables.

**CONCLUSIONS:**

Implementing the patient’s preoperative clinical evaluation to reduce the occurrence of related last-minute cancellations might mitigate its negative impact on survival.

## Graphical abstract

**Figure:**
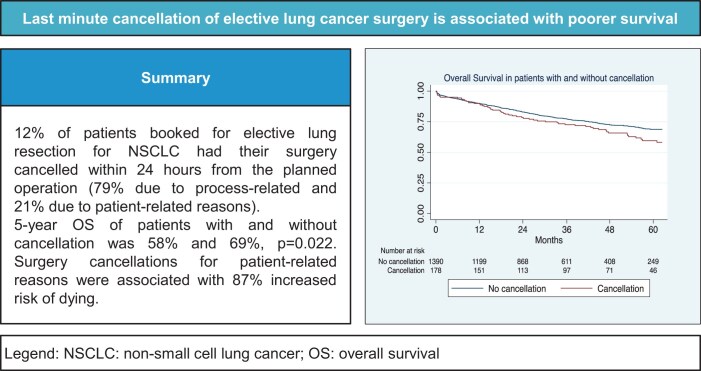

## INTRODUCTION

Previous studies have highlighted the negative impact on patients’ and relatives’ psychological status following last-minute cancellations (LMCs) [1] and its relationship with poorer prognosis [1–5]. As practising surgeons, we have direct empirical experiences of the potentially devastating consequences of a LMC on our patients, who lose trust in the process, feel unsafe going home without having received treatment and experience the uncertainty of future rescheduling. In addition, cancellations have severe negative effect on the institution itself, with resources waste (i.e. operating room underutilization), increasing waiting list and additional costs (i.e. need for repeating out-of-date investigations) [4–6]. Finally, the cancellations of elective procedures have an impact on the training of the surgical residents: in the context of a public healthcare system where the increasing service provision demands, and the scrutiny of working hours have already reduced the number of procedures performed by the individual trainee by the end of her/his training [5], the patient’s cancellation is a missed training opportunity.

Despite these considerations and despite Wong *et al.* [3] describing a high rate of overall surgery cancellation in the British National Health System, as high as 13.9%, there are no recognized standard operating procedures to prevent a surgical cancellation and to investigate it when it happened.

Therefore, we set this study with the objective to analyse the prevalence of LMCs, specific to curative-intent surgery for lung cancer at our institution, to investigate the causes of the cancellations and finally to discover their association with patients’ prognosis. The main rationale was to generate new evidence to ultimately raise awareness in our specialty and generate additional studies to inform standard operative procedure to investigate and prevent surgical cancellations [6–10].

## PATIENTS AND METHODS

### Study design

This is a single centre, retrospective, observational cohort study. We retrospectively analysed consecutive patients booked for elective lung cancer curative-intent resections.

### Ethical statement

The Institution Review Board of Leeds Teaching Hospitals reviewed this study and considered it as service evaluation not requiring formal review by the research ethics committee, and individual patient’s consent for this retrospective analysis was waived.

### Patients

Patients’ data were gained by searching the bespoke database of the Department of Thoracic Surgery, St James’s University Hospital, Leeds, UK, from 1 January 2017 and until 31 December 2022. Patients booked for procedures other than lung cancer resections with curative intent were excluded. Within this group (patients who were admitted to the department to receive lung cancer curative-intent surgery), we identified patients who sustained a LMC; this was defined as a cancellation occurring within the last 24 h from the planned operation time as per Scheenstra *et al.* [2].

These patients were identified from the operating theatre list records, and the reason for cancellation was specified in the theatre list, and/or on the bespoke electronic system, and/or in the patient records.

The following variables were retrieved and analysed along with the presence and type of cancellation: gender, age, body mass index, forced expiratory volume in 1 s expressed in percentage of predicted for age and gender, carbon monoxide lung diffusion capacity %, history of coronary artery disease and cerebrovascular disease, Eastern Cooperative Oncology Group Performance score, American Society of Anesthesiology score, clinical staging, surgical access (open versus minimally invasive), extent of resection (wedge, segmentectomy, lobectomy, pneumonectomy). Most of the variables were complete. Carbon monoxide lung diffusion capacity was missing in 2 patients. The missing value was replaced with the mean of the non-missing values. For every cancellation, it was investigated whether the surgery was rescheduled or not; the incidence of rescheduling in each cancellation category was reported. For every rescheduled procedure, the delay between cancellation and rescheduling was evaluated in every cancellation category. Ninety-day mortality and the 5-year overall survival (OS) were investigated and relation with cancellation were reported. The follow-up started from the date of cancellation only for those patients who were not rescheduled.

### Categories of cancellation

The recorded reasons for cancellation were analysed and were then grouped into 2 main categories: process-related (PR) and patient-related (PTR) cancellation. PR cancellation was attributed when the leading factor that led to the cancellation was related to a hospital process problem. PTR was attributed when the leading factor that triggered the cancellation was a medical reason or patient’s choice, i.e. patient’s new condition, change in treatment plan, etc.

### Statistical analysis

A descriptive statistic was performed to report the prevalence of LMCs, and their reasons were reported. Numeric variables with normal distribution were compared between categories using the unpaired Student’s *t*-test, while those without normal distribution were compared using the Mann–Whitney *U*-test. Categorical variables were compared between groups using the Chi-squared test or the Fisher’s exact test (in case the number of observations was <10 in at least 1 cell). Follow-up information was obtained by data retrieved from the hospital electronic clinical information system. All patients were followed up through October 2023. No patient was lost at follow-up. OS was measured from the date of surgery to the date of death from any cause and censored at the date of data retrieval for survivors.

Survival end-points were characterized with the use of the Kaplan–Meier estimator. A log-rank test was used to estimate the *P*-value for testing differences between the category of cancellations, modified with Bonferroni correction.

Hazard ratios and their confidence intervals (CIs) were estimated with the use of Cox proportional hazards models with robust indicators. A stepwise backward elimination of variables was performed with *P* < 0.1 set as threshold for variable retention in the final regression model. Along with the presence and type of cancellation, the following variables were initially used as independent co-variates in the regression model based on their clinical relevance: age sex, body mass index, forced expiratory volume in 1 s, carbon monoxide lung diffusion capacity, clinical T stage, clinical N stage, presence of coronary artery disease, ASA score, Charlson’s comorbidity index, surgical access (open versus minimally invasive) extent of operation (wedge resection, segmentectomy, lobectomy, pneumonectomy), no surgical treatment. Patients who did not receive surgery after being cancelled were still included in the analysis, and a co-variate was created to indicate their definitive treatment (surgery versus no surgery) to adjust the analysis accordingly, as not having surgery may have influenced the outcome within those who had a cancellation.

The surgical delay predictor was tested for a possible association with survival using three-knots restricted cubic splines in a Cox proportional hazard model.

All tests were performed using the Stata 15.0 statistical software (Stata Corp, College Station, TX, USA).

## RESULTS

### Patients’ characteristics and reasons for cancellation

Between January 2017 and December 2022, 1587 patients were scheduled for thoracic surgery with the indication of pulmonary malignancy curative-intent surgery. One-hundred and ninety-seven patients of 1587 (12%) sustained a LMC; 156 (79.1%) were PR and 41(20.8%) were for PTR cancellations.

Twenty-one (11%) of the cancelled patients never received surgery; 3% (5/156) of PR cancelled patients did not receive surgery and 39% (16/41) of those cancelled for PTR reasons did not receive surgery, *P* < 0.0001.

Comparison of patients’ characteristics is summarized in Table 1. There were no significant baseline differences between the 3 groups (non-cancelled, PR and PTR). There were no significant differences in patients’ comorbidities, fitness test and performance status. The American Society of Anesthesiologists score was higher in the patients who were cancelled: ASA was higher than 2 in 318/1390 (23%) in the non-cancelled group, in 63/146 (40%) in PR and 22/41 (54%) in PTR group.

**Table 1:** Comparison of patient-related characteristics between different cancellation status

	No cancellation (no. 1390)	Process-related (no. 156)	Patient-related (no. 41)	ANOVA *P* value
Age	69.0 (9.0)	68.8 (10.9)	69.2 (8.3)	0.96
Gender (male), *n* (%)	643 (46%)	66 (42%)	21 (51%)	0.51
BMI (kg/m^2^)	27.2 (5.3)	26.7 (5.2)	26.6 (5.0)	0.50
FEV1%	90.3 (21.1)	89.0 (22.1)	84.0 (20.0)	0.09
DLCO%	75.8 (18.8)	73.7 (18.9)	73.4 (16.5)	0.31
CCI > 1 (n,%)	367 (26%)	49 (31%)	14 (34%)	0.24
ASA > 2 (n,%)	318 (23%)	63 (40%)	22 (54%)	<0.001
CAD (n,%)	145 (10%)	15 (9.6%)	3 (7.3%)	0.78
Diabetes	180 (13%)	19 (12%)	3 (7.3%)	0.60
PS > 1	125 (9.0%)	18 (12%)	4 (9.7%)	0.58
CVD	53 (3.8%)	4 (2.6%)	3 (7.3%)	0.33

Results are expressed as means and standard deviations for numeric variables and count and percentages for categorical ones.

ASA: American Society of Anaesthesiologists score; BMI: body mass index; CAD: coronary artery disease; CCI: Charlson’s comorbidity index; CVD: cerebrovascular disease; DLCO: carbon monoxide lung diffusion capacity; FEV1: forced expiratory volume in 1 s; PS: performance score.

The detailed reasons for cancellation per group are shown in Table 2. The most frequent reason for cancellation in the PR group was theatre unavailability due to previous cases overrun in 42/156 patients (27.5%). The 2nd most important reason in this group was hospital beds unavailability in 40 cases (26%) followed by staff unavailability (surgeons, nurses or anaesthesiologists) in 23 cases (15%).

**Table 2:** Detailed causes of cancellation in each group—hospital-related and patient-related group

	No. patients (%)
Hospital-related cancellations	
ICU/HDU bed unavailable	40 (26%)
Ward bed unavailable	21 (14%)
Urgent case took priority	16 (10.5%)
Theatre overran	42 (27.5%)
Management change	7 (4.5%)
Staff unavailable	23 (15%)
Theatre ventilation failure	3 (1.9%)
Admin error	1 (0.6%)
Patient-related cancellations	
Deemed unfit for surgery	26 (65%)
Patient’s decision	8 (20%)
Additional tests required	6 (15%)

ICU: intensive care unit; HDU: high dependency unit.

In the PRT group, we identified only 3 themes for cancellation: the patient was deemed unfit on the day by surgeon or by the anaesthesiologist in 26 cases/41 (65%), the patient changed mind regarding her/his own treatment in 8 cases (20%) and additional tests were requested in 6 cases (15%).

### Patients’ outcomes

The 90-day mortality rate was similar between cancelled and non-cancelled patients: 9/197 (4.6%) vs 66/1390 (4.7%), *P* = 1. The 90-day mortality rate of those patients who were cancelled for PTR reasons was 4.9% (2/41) vs 4.5% (7/156) in those cancelled for PR reasons (*P* = 1).

Fifty of 197 (25%) cancelled patients were T upstaged from T1 to >T1 (vs 243/1390, 17% of non-cancelled patients) (*P* = 0.007). Fifty-eight of 197 (29%) cancelled patients were N upstaged from N0 to N1 or N2 (vs 277/1390, 20% of non-cancelled patients, *P* = 0.002).

**Figure 1:**
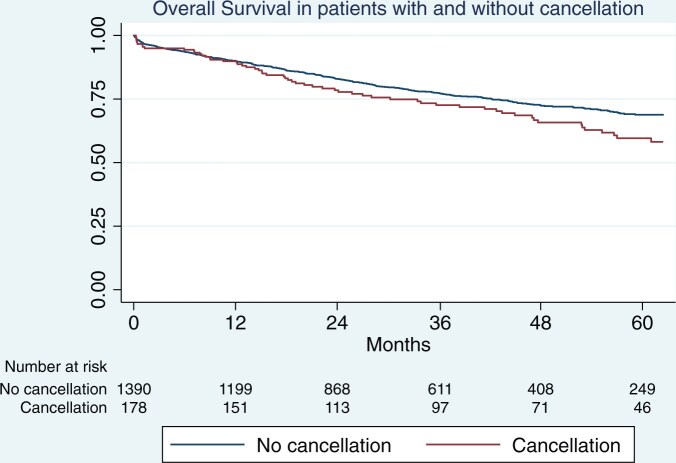
Overall survival in patients with and without cancellations.

**Figure 2:**
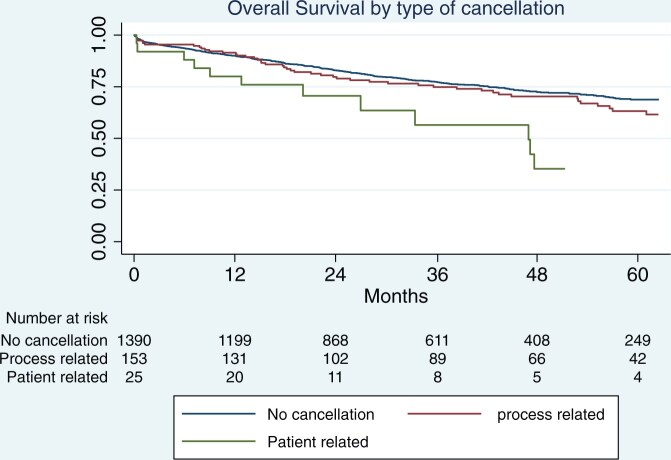
Overall survival by type of surgical cancellation.

The follow-up index in all 3 groups was 1 as we did not have any patient lost to follow-up. Median follow-up in patients without cancellation was 41 months (IQR 10–83). It was 48 months (IQR 9–82) and 38 months (IQR 14–81) in those with a PR and PTR cancellation, respectively. Five-year OS in patients without cancellation was 69% (95% CI 66–71) vs 58% (95% CI 49–66) among those who had a cancellation (Fig. 1). The difference in survival between these 2 groups was statistically significant (*P* = 0.022). In particular, the 5-year OS of those cancelled for organizational reason was 61% (95% CI 52–60) vs 35% (95% CI 14–58) in those cancelled for PTR reasons (adjusted *P* value for multiple comparisons = 0.14). On the other hand, there was no difference in survival between patients without cancellation (69%, 95% CI 66–71) and those with PR cancellation (63%, 95% CI 54–71) (adjusted *P* value for multiple comparisons = 0.33). The survival of patients with PTR cancellation was significantly poorer than those without cancellation (adjusted *P* value for multiple comparisons = 0.0072) (Fig. 2).

The median survival for those with process-based cancellation was 74 months versus 48 months in those with PTR cancellations.

Cox regression analysis showed that surgery cancellation within the last 24 h for PTR (hazard ratio 1.87, 95% CI 1.02–3.42, *P* = 0.043) reasons remained a factor associated with poorer OS after adjusting for clinical stage, type of operation, and PTR variables (Table 3).

**Table 3:** Results of the Cox regression analysis with robust indicators to assess the association of surgery cancellation and overall survival

Variables	HR	*P* value	95% CI
Age	1.03	<0.001	1.02–1.05
Gender male	1.51	<0.001	1.23–1.86
DLCO	0.98	<0.001	0.97–0.98
Clinical T stage > 1	1.64	<0.001	1.31–2.05
Open approach	1.83	<0.001	1.42–2.37
Process-related cancellation (ref. no cancellation)	1.22	0.17	0.91–1.63
Patient-related cancellation (ref no cancellation)	1.87	0.043	1.02–3.42

Parsimonious model (only variables with *P* < 0.1 after backward stepwise elimination are shown).

CI: confidence interval; DLCO: carbon monoxide lung diffusion capacity; HR: hazard ratio.

### Outcomes by treatment’s delays and cancellation status

The median delay from cancellation to surgery was 12 days (5–21 days range) for process-based cancellations and 16 days (10–35) for PTR reasons, *P* = 0.012 (Table 4). The median overall delay was 13 days.

**Table 4:** Outcomes according to cancellation status

	No cancellation (no. 1390)	Process-related (no. 156)	Patient-related (no. 41)	*P*-value
Rescheduled surgery, *n* (%)		151 (97%)	25 (61%)	<0.001
Days till surgery, median (IQR)		12 (5-21)	16 (10-35)	0.012
30-day deaths	48 (3.4%)	5 (3.2%)	2 (4.9%)	0.87*
1-year deaths	138 (9.9%)	13 (8.3%)	5 (12%)	0.70*
3-year deaths	276 (20%)	35 (22%)	9 (22%)	0.66*

Results are expressed as count and percentages of total within each category.

* *P*-test for trend, no significant differences were detected between individual categories (*P* > 0.5 in all inter-group comparisons).

The surgical delay from cancellation was not associated with poorer OS when tested in a Cox proportional hazard model using three-knots restricted cubic splines. Table 4 reports the outcomes according to cancellation status. We were not able to find any difference in 30-day, 1- or 3-year mortality between groups.

## DISCUSSION

As previously published literature suggested that LMC is associated with poorer clinical outcome, the main objective of this study was to investigate if this applies to lung cancer surgery. Consequently, we aimed to identify modifiable risk factors (PTR or hospital-related) for cancellation that demands systems’ implementation.

### Main findings

The OS difference was statistically significant between cancelled patient and non-cancelled patient, and it was also significant between hospital-related cancellation and PTR cancellation. There was no difference in age, gender, respiratory function tests or performance status in the 3 groups (no cancellation, PR and PTR cancellations). ASA score was higher in cancelled patients, but we could not identify other independent risk factors for cancellation, unlike other studies [2].

The PTR cancellation group is associated with poorest prognosis. The main reason for cancellation, in this group, was that patient was deemed unfit for general anaesthetics or surgery on the day (26 patients/41, 65%). Within the reason of being unfit, ongoing infection, anaemia, neutropenia and new symptoms being recurrent themes. The 2nd most relevant group within the PTR cancellation, that accounted to 20%, are patients who cancelled themselves. These 2 types of events may be mitigated by an additional review, closer to the date of surgery, and more extensive pre-habilitation and lung surgery pre-operative counselling, respectively [8, 11, 12].

There was statistically significant difference in the rescheduling rate of patients between the 2 groups of PR and PTR cancellations: operations that were cancelled for PR reasons were rescheduled in 96.7% of the cases and with a median of 12 days delay.

Operations that were cancelled for medical reasons were only rescheduled in 60.9% with a longer delay (Table 2) *P* < 0.0001. In the patients who were rescheduled, there was not statistically significance in the delay time between the 2 groups.

During 30 days of follow-up, 48 deaths (3.4%) occurred in the non-cancellation group, 5 (3.2%) occurred in the PR group and 2 (4.9%) deaths occurred in the PTR group and these were not statistically significant, *P* = 0.87. PR-related cancellations were mainly related to operating theatre availability (previous case overrun), staff shortages/bed availability and emergencies who took priority.

System implementations are required for better theatre list scheduling [13] as the previous case overrun was a recurrent theme and was potentially preventable. A proportion of complex cases that may require a longer procedure can be identified in advance, hence discussed at high-risk multidisciplinary meetings. Planning of anaesthetic intervention and dual specialist attendance may be cost-effective measures if they result in lower cancellation rate of the subsequent scheduled procedures and need to be further evaluated [14, 15].

The 2nd main theme in PR cancellation is bed availability. An expansion of inpatient beds is complex but maximizing the outpatient procedures and the day cases services would create more bed availability, while segregation of elective and urgent services would ring-fence both theatre access and bed availability for scheduled patients [16].

### Limitations

Our study was an observational, retrospective patients’ cohort study and therefore the reason for cancellations was sometimes associated with limited recorded information. However, the main reason for cancellation could be identified in all cases. As currently there are no standard operative procedures in place to prevent or investigate a cancellation, the records on reason for cancellation were heterogeneous and subjective.

This study was also a single-centre cohort, executed in a large tertiary academic referring centre that performs a large volume of lung cancer surgeries per year. Therefore, this study may be a good representation of similar thoracic surgery unit within the country or within similar public health care system (National Health System) but may not represent the reality of substantially different health care systems.

It may be probable that the study was underpowered to detect clinically relevant differences between categories of cancellation. This is especially considering the small number of patients in the PTR group. This should be considered when interpreting the results.

Finally, the interval between clinic consultation and surgery was not recorded; this may significantly correlate to PTR cancellations, as a longer interval may allow more times for clinical changes to happen while awaiting surgery.

## CONCLUSION

Unanticipated LMCs of elective lung cancer surgery are common and are associated with poorer oncologic outcomes. We believe that our results may contribute to raise awareness on this problem and on its impact on outcome. Our findings would assist in the implementation of patients’ workflow with a view to minimize surgery cancellation, mitigate their negative impact on cancer prognosis, and optimize hospital resources. Prospective studies will be required following implementation of clinical and structural processes.

## FUNDINGS

No funding was available for this study.

**Conflict of interest:** Dr Alessandro Brunelli received consultancy fees for speaker honoraria and advisory Board roles with Astra Zeneca, BMS, Ethicon, MSD and Roche. No conflicts of interest disclosed by other authors.

## Data Availability

The data underlying this article will be shared on reasonable request to the corresponding author.

## ABBREVIATIONS

CI: Confidence interval
LMC: Last-minute cancellation
OS: Overall survival
PR: Process-related
PTR: Patient-related
